# A new contribution to research metrics

**DOI:** 10.1080/03009734.2018.1542644

**Published:** 2018-12-04

**Authors:** Arne Andersson, Joey Lau Börjesson

The last issues of our two latest volumes have both contained editorials dealing with the performance of *Upsala Journal of Medical Sciences* (UJMS) in terms of numbers of submissions and citations of articles. Thus, we have reported on the decline of the number of submitted manuscripts, most probably reflecting the extensive appearance of so-called predatory journals (1–3), but also on the joy of announcing the passage for the first time of the critical 2.0 level of the much-discussed impact factor (4, 5). In that last report we dwelt on how to maintain or even increase these impact figures. This is an attempt to brief you on the progress of that proposal.

**Figure 1.**
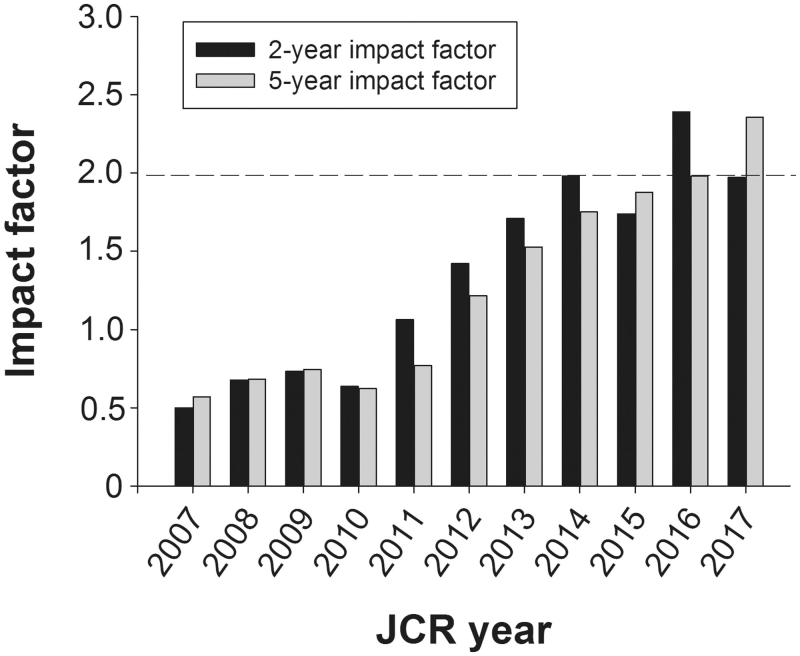


Perhaps it would be most suitable at this time point to inform you on the latest impact factor figures from Clarivate Analytics in June, last summer, before discussing the proposed moves. We have to announce a slight drop—from 2.389 to 1.971—of the 2-year impact factor figure (Figure 1). That most likely illustrates the fate of many smaller and not frequently published journals in that single articles have a very strong impact on the value, resulting in a fairly bumpy ride. Quite in contrast, the 5-year factor for UJMS has been constantly growing for more than 10 years. For the sake of clarity, this factor is calculated after introducing in the denominator the number of documents the last five years before the calculation year. This year, we reached a record high 2.355 (Figure 1). And more will come. That is what can be seen from the preliminary figures of this year.

In the year 2016 editorial (1) we discussed the fate of individual articles in terms of citation figures. We then determined the number of articles belonging to the 99th percentile most-cited papers during a specific year (year three after the publication). For the period 2010–2014 with 227 publications, three papers qualified for the 1% (more than 21 cites) and 23 for the 10% category (more than 7 cites). During the two following years one was identified as a ‘1% cited paper’ and six more as belonging to 10% category. Thus, highly cited papers show up as frequently these days as before. The most cited paper by Anders Larsson et al. (6) on ‘The state of point-of-care testing’ is another piece of evidence that review articles tend to become most cited. One of the 10% articles, the Rudbeck Award review by Lena Claesson-Welsh (7), illustrates the fact that papers published late in the year are very much hampered by the shorter exposure time in year 1. In her case the citation figure, 15 for years 2015–2017, has more than doubled at this time point in the year 2018.

**Figure 2.**
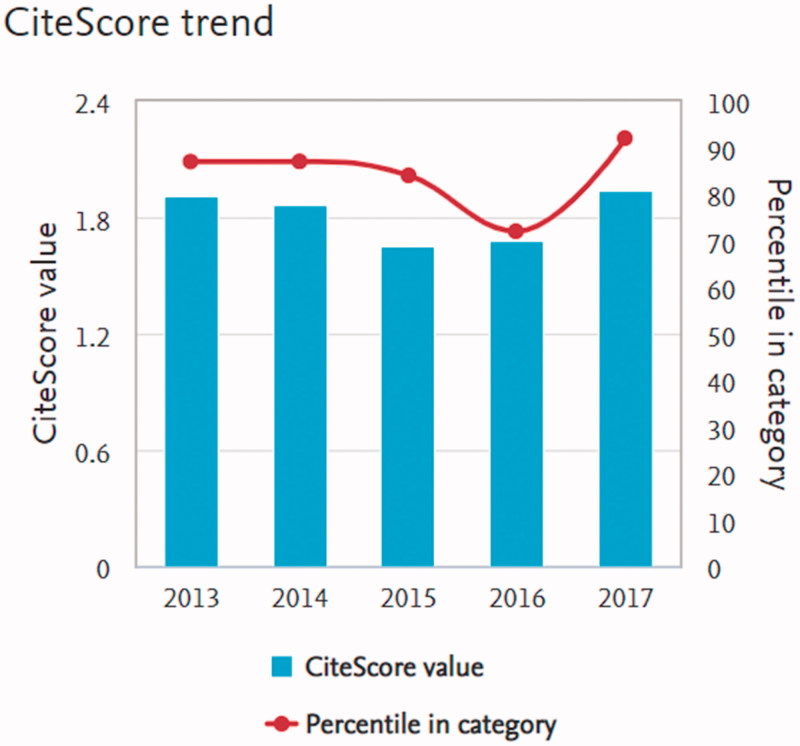


Interestingly, Scopus has recently launched an alternative research metric besides their SJR and SNIF scores, the so-called CiteScore (8). It measures the average citations per document published within three years ahead of the year for the calculation of the pertinent CiteScore. In other words, one more impact factor estimate, but now on a three-year basis. It, however, differs from the traditional Clarivate metric in that it includes all available document types. Thus, case reports, letters, editorials, commentaries, etc. will all be added in the denominator. To that should be added the fact that the Scopus database contains about 50% more journals. As a consequence of that, CiteScores for most biomedical journals will be considerably lower than the Clarivate impact factor value. This difference is perhaps most evidently displayed for the *New England Journal of Medicine*: 79.830 is their 2017 Clarivate score and 14.75 the CiteScore. A more than five-fold decrease when all categories of published documents have to be taken into account. Likewise, the Lancet had a six-fold drop from 53.254 (Clarivate) to 8.60 (CiteScore). It is worthy of note that the corresponding difference for UJMS is close to zero, since our CiteScore is as high as 1.94 for the year 2017 (Figure 2).

This new research metric also offers a so-called CiteScore Tracker for the year under evaluation. This gives everyone an opportunity to continuously monitor the increase of the CiteScore value, since it is updated monthly. At present, in mid-October, the value for UJMS is above 1.60, suggesting a considerable increase compared with last year. There is also a rank system for journals belonging to different research fields. UJMS has become sorted into the General Medicine category that consists of 841 journals. Our rank position is 61 corresponding to the 92th percentile (Figure 2). The corresponding figure for UJMS in Clarivate rank for that group of journals is 54 out of 155. The most obvious explanation for this discrepancy most probably is the inclusion of more non-prestigious journals in the Scopus database.

**Figure 3.**
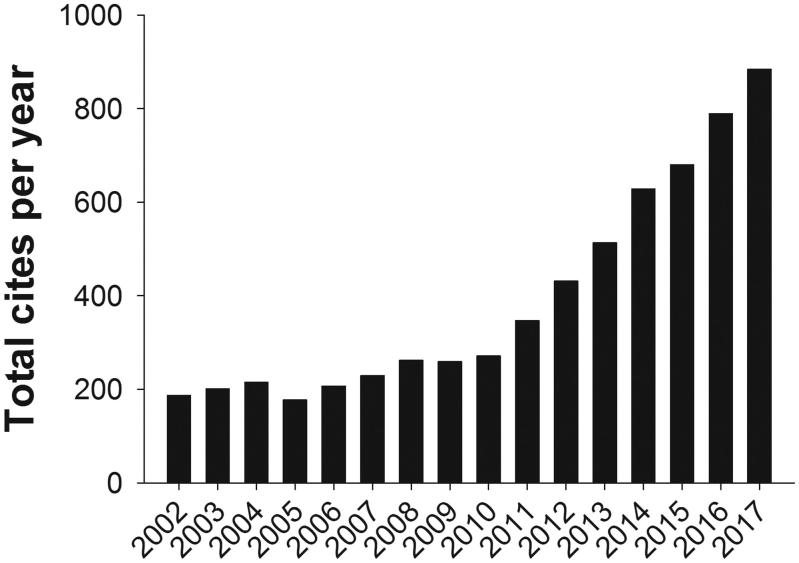


One more citation figure worthwhile to mention is that for the number of total cites (Figure 3). This is perhaps the most important value to follow. The figure shows one more year with a substantial increase—about 10%—for UJMS that cannot simply be explained by the fact that one more volume is out there to be cited and also many more journals active in scholarly publishing. I can remember when reporting to our board of the Upsala Medical Society some years ago when our cooperation with Taylor & Francis had just commenced that we then proudly could claim that each day there was a new citation of a UJMS article. Now, this figure is rapidly approaching three new citations a day.

Over the last few years we have faced decreasing numbers of submissions. Actually, last year we had to report an ‘all-time low’ with only 175 submitted papers. When this is being written (mid-October) that number has already been passed. Preferably, one would like to interpret that as if our measures presented a year ago have been effective—free color-prints, fast-track opportunities (admittedly, we have not had many), free printed issues distributed to all society members, and maintenance of the ‘free-of-charge’ system despite our open-access publishing. Perhaps we could have exaggerated our presence on social media. For an old-fashioned editor the efficiency of the work load is difficult to estimate, but many high Altmetric scores (9) are noteworthy and rewarding. Therefore, in this context we have to rely very much on you, readers and authors.

One more measure to facilitate and convince potential authors to choose UJMS for their next submission is that of ‘format-free submission’. That is supposed to make the process of preparing research papers for submission simpler and less time-consuming. The post-acceptance reformatting will be, as much as possible, managed by our publisher. Hopefully, this will ensure that the research of the authors can be disseminated quicker through a speedier publication process. By such means we should be able to attract high-quality papers, a prerequisite for remaining successful in times of impactitis (10, 11).

Finally, we mentioned last time that special issues have been very valuable when discussing how to improve the impact of our journal. The next to follow—that from Uppsala Clinical Research Centre—will appear as the first issue of next year. The two most recent ones, denominated ‘Remembering Claes Hellerström’ (12) and ‘Preconceptional Health and Care’ (13) have been very successful in terms of citations, and to a great extent they can explain our fairly high citation figures. I will take this opportunity to invite researchers, who want to present their research as well as that of excellent colleagues both from their own research environments and abroad, to edit such a special issue. We can offer quick and efficient editorial assistance. We are looking forward to a most pleasant collaborative effort for the sake of scholarly publishing in general and our old and venerable journal in particular.
